# Revision of instructions to authors for pharmacology research and perspectives: enhancing the quality and transparency of published work

**DOI:** 10.1002/prp2.106

**Published:** 2015-01-30

**Authors:** Michael J Curtis, Darrell R Abernethy

## Abstract

e00106

Over at least the past two decades, there have been calls for improvement in the conduct and reporting of nonclinical and clinical research in general, and more specifically in the pharmacological sciences. These calls have recently become more strident with reports of lack of reproducibility of research results that are in the published literature (Prinz et al. 2011). This has stimulated activity on both sides of the Atlantic (and beyond) for improved experimental design and improved reporting of research results of pharmacology studies (Landis et al. 2012; Mullane et al. 2013; Collins and Tabak 2014; Curtis et al. 2014; McNutt 2014). Unfortunately, the real and perceived inadequate performance and reporting of basic and clinical pharmacology research studies have continued. Standards for reporting of clinical trials, including pharmacology studies (the CONSORT standards) were first outlined in 1996 (Altman 1996) and have been periodically updated since that time (Moher et al. 2001, 2010; Schulz et al. 2010). Based on evidence that the CONSORT standards have improved the conduct and reporting of clinical research, the ARRIVE guidelines that are based on the same principles for conduct and reporting of animal research studies have been elaborated (Kilkenny et al. 2010).

To address issues of quality studies and reporting of their finding for this journal, we have updated the instructions to authors for submissions to Pharmacology Research & Perspectives. As we entertain publication of both nonclinical and clinical studies in the pharmacological sciences, the core components of both the ARRIVE guidelines and CONSORT standards are embodied in the revised instructions to authors. Key elements that are now explicitly incorporated in the PR&P instructions to authors are more extensively stated in Curtis et al. (Curtis et al. 2014), however, they incorporate the following principles.

1. Justification of sample sizes, including analysis of statistical power should be in the Methods section. Sample sizes should be at least *n* = 5 per control and experimental arms.
2. Experimental and control groups should be of equal size, or if they are not, a scientific justification for why they are not should be presented (e.g., clinical case–control study).
3. Randomization of study units (individual animals, in vitro study preparations, or people) should be conducted, with exclusion and inclusion criteria clearly defined. If exclusions are replaced to maintain sample size the randomization should be maintained. If randomization is not part of the study design, a scientific justification for why it is not should be presented.
4. Individuals making the experimental observations should be blinded to treatment or group assignments. In the case of clinical studies, study subjects should also be blinded to the treatment (double blind design). If blinding is not part of the study design, a scientific justification for why it is not should be presented.
5. Normalization of data (e.g., to account for baseline differences) is discouraged, and if done, it must be scientifically justified and the methods used clearly described.
6. For statistical group comparisons, the threshold for statistical significance (*P* value) should be prospectively identified and defined in the Methods and not varied in the data analysis. Similarly, the hypothesis being statistically tested should be prospectively identified and defined in the Methods.

The Methods section must completely and thoroughly characterize the approaches used to derive the data and the statistical methods being presented in the manuscript, and this should be in sufficient detail for an experienced scientist to replicate the findings. We refer you to the referenced work cited below, and believe that adherence to the revised Instructions to Authors for PR&P should be useful to address the concerns about data reproducibility that are currently in the scientific community.
